# Rape on an Idiot

**Published:** 1854-11

**Authors:** 


Rape on an Idiot.—In the Court of Common Pleas of Athens County, Ohio. March Term, 1853. Before Mr. Justice Nash.

State of Ohio against John Crow.

A female *idiot*, or an *insane female*, may be the subject of a rape.

An assault with an intent to commit a rape may be committed on the person of an insane female, or a female idiot.

The crime of having carnal knowledge of an insane female, knowing her to be such, is consummated when the act is done knowingly with her acquiescence or consent.

The word insane in the sixth section of the act for the punishment of crimes, Curren's *Revised Statutes*, pp. 184, 185, is used in its elementary and popular meaning, unsoundness of mind; and hence embraces the case of one's having carnal knowledge of a female idiot, knowing her to be such.

The defendant was indicted: 1, for having committed a rape on the person of Louisa Dowler; 2, for an assault with intent to commit a rape on the said Louisa; and 3, for having had carnal knowledge, she, said Louisa, being an insane woman, and he, said defendant, knowing her to be such. The defendant pleaded not guilty, and the cause was tried by a jury at the last March Term of the Common Pleas.

The evidence on the trial proved that said Louisa Dowler was of unsound mind, and had been so from her nativity: though she was not so absolutely destitute of mind that she did not perform the necessary functions and calls of humanity; but that she had not mind enough to testify as a witness, or to be held legally responsible for her acts, whether civil or criminal.

The words of the statute are: "That if any male person, seventeen years old and upwards, shall have carnal knowledge of any other woman than his wife, such woman being insane, he knowing her to be such, every person so offending shall be deemed guilty of a misdemeanor, and upon conviction thereof, shall be imprisoned in the penitentiary, and kept at hard labor not more than ten, nor less than three years."

Mr. *Kowles* for the State.

Messrs *Nye* and *Jewett* for the defendant, claimed that the said Louisa, being an idiot, had no will, and, therefore, that a rape could not be committed on her person against her will; it was further claimed, that the word insane, in the sixth section of the act, did not embrace an idiot; and hence, that the defendant could be convicted of neither of the charges embraced in the indictment.

Mr. Justice Nash. It is claimed, *first*, that a female idiot is not the subject of a rape; that she has no will, and hence, an act cannot be done to her person against her will. No authorities are cited for this startling position. On looking into the books, I can find no such distinction intimated; and, if such was the law, it is singular that so important a qualification of the crime of rape should not have been noticed hitherto in any treatise on this subject. Rape is defined to be, the having carnal knowledge of a female, *forcibly and against her will.* There is here no limit to the use of the word female; nothing said as to the soundness or unsoundness of her mind, as to idiocy or insanity. In this respect our statute follows the common law; and must, therefore, be construed as the same words were construed in the definition of the crime at common law.

There is another consideration not to be overlooked. The section providing for punishing assaults with a criminal intent, declares that an assault committed on another, with the intent to commit a rape, shall be criminal. Now, if a rape cannot be committed on the person of an idiot, then it is no crime to assault her person with such an intent. The same question applies also to assaults committed on an insane person; since this argument places them without the protection of the law, punishing the crime of rape. Nor are insane persons protected under the sixth section, since the crime there described is committed only when the perpetrator knows the woman to be insane. Indeed, that section is clearly limited to the case of a male person's knowingly having sexual knowledge of an insane female without resistance on her part, and with her acquiescence. Hence this section cannot be made to embrace the case of one having such sexual intercourse forcibly and against the will or resistance of such insane female.

It is further claimed that an idiot is not an insane person under the meaning of that term in the sixth section. This result then follows, that a female idiot is left wholly unprotected against this class of crimes. A person cannot be punished for having carnal knowledge of her person forcibly and against her will, as she has no will to overcome; she is not an insane person, and so not under the protection of the sixth section, and neither an idiot nor an insane female is protected against the assaults made with an intent to commit a rape, since a rape cannot be committed on the person of either.

It must require some very cogent reasoning, or some very convincing authorities, before the court could be induced to give a construction to a statute which must lead to such results. But here is no such authority; no such decision has been found. Is there any more force in this reasoning? Let us examine it for a moment.

In the first place, where the carnal knowledge is had by *force*, it must be against the will of the female. Nor need there be any direct evidence of this action of the will; the law implies the want of consent from the force itself. It is the *consent* of the female which takes away all criminality from this connection; it is this want of consent which renders this connection obtained by force, criminal. Hence, if an idiot has no will to be overcome, she has none to consent; and the law implies that the act being accomplished by force, is done against her will.

But is it true that an idiot or insane person has no will? What is the definition of these two words? Do they imply the *loss* of *will*, or a mere *unsoundness* of mind? These words are thus defined by Webster: "Idiot—a natural fool, a fool from birth; a human being in form, but destitute of reason or the ordinary intellectual powers of man. Insane, unsound in mind or intellect; mad; deranged in mind;" and one of the words used to define *insanely* is *foolishly*. Fool is defined to be one who is destitute of reason, or the common powers of understanding; an idiot. Some persons are born *fools*, and are called *natural fools*; others may become *fools* by some injury done to the brain. In Chitty's *Medical Jurisprudence*, p. 348, "an idiot is defined to be a person who has been *defective* in intellectual powers from the instant of his birth, or at least before his mind had received the impression of any idea." Again, Chitty says, "that idiocy consists in a defect or sterility of the intellectual powers; but it may be induced in after life; while lunacy and madness consists in a *perversion* of intellect." All these definitions imply either a *weak ness*, or *perversion* of the mind, or its powers, not their *destruction*. The powers are still all present, but in an impaired and weakened state. Hence, an idiot cannot be said to have no *will*, but a *will weakened* and *impaired*—a will acting, but not acting in conformity to those rules, and motives, and views, which control the action of the will in persons of sound mind. Indeed, in an insane person, the will is often too fearfully active, and wholly uncontrollable by reason or persuasion. There is here no lack of will, but simply a *perversion* of it. Nor is this the most conclusive answer to this argument. If there is no will how are the voluntary actions continued? Actions, which, like respiration, are instinctive, are independent of the will; but eating, and numerous other acts, which necessarily imply the exercise of the will, are performed by idiots and insane persons; and their exercise demonstrates the existence of a will; of a will which can assent to, or dissent from, what are clearly voluntary acts. I have, therefore, no hesitation in holding that both idiots and insane persons are possessed of a will, so that it may be legally and metaphysically said, that a carnal knowledge may be had of their persons *forcibly* and against their will.

The next inquiry is, what is the proper construction to be given to the word *insane?* In the sixth section of the act for the punishment of crimes, *Curren's Revised Statutes*, p. 184, that section provides: "That if any male person, seventeen years old and upward, shall have carnal knowledge of any woman other than his wife, such woman being *insane*, he knowing her to be such, shall be deemed guilty," &c. It is claimed that this word *insane* does not embrace a female who is an idiot. We have already seen that idiocy may be induced after infancy, as well as be congenital, Chitty's *Med. Jurisprudence*, p. 347, and that both terms are defined by the same words, *unsoundness of mind.* In the one case, this unsoundness of mind develops its existence in want of capacity to reason at all; or, at least, in a much less degree than the generality of mankind; while, in the other, there is, perhaps, greater acuteness, though upon false and fancied hypothesis, Chitty's *Med. Jurisprudence*, p. 348. Still, in both cases, unsoundness of mind is the cause. The very origin of the word *insane* demonstrates this; in its Latin origin, it is a word simply meaning *unsound*, and nothing more; and in the popular language it is used in this sense to this day, whatever may be the specific meaning attached to it by writers on mental diseases. If, then, the object and policy of this statute embraces idiots as well as lunatics, there is nothing in the use of the word *insane* which absolutely precludes us from giving that elementary meaning to the word in this statute. The reason of this provision clearly applies to idiots, as well as to lunatics; if there is any reason in the case of female lunatics, why sexual intercourse with them should be prohibited, equally strong is the reason why it should not be permitted with female idiots. If the offspring in the one case might be affected by insanity, so in the other it might be with idiocy. Whatever reason, therefore, can be found to call for the law in relation to female lunatics, will apply in any equally cogent manner to idiots. If the one class ought to be protected, equally so ought the other.

Such then being the manifest scope of the law, I can have no hesitation in concluding that such was the intention of the legislature; that this word *insane* was used in its elementary and popular meaning, as descriptive of that unsoundness of mind which renders individuals civilly and criminally irresponsible for their acts, whether that unsoundness discloses itself in idiocy or lunacy.

In accordance with these views, I hold that a female idiot or an insane female is the subject of a rape; and hence of an assault with the intent to commit that crime; and that a male person, of a proper age, who shall have carnal knowledge of a female idiot, knowing her to be such, is guilty under the sixth section of having carnal knowlege of an insane woman, knowing her to be such.

The jury were so charged, and they returned a verdict of guilty of an assault with an intent to commit a rape, and not guilty of on the other two counts. And sentence was passed on the prisoner.—*Western Law Journal*, vol. x. pp. 501–5. T. R. B.

